# Evaluating the Effects of Self-Monitoring of Performance with a Peer Component on Disruptive Behavior and Task Completion of Children with Autism Spectrum Disorder

**DOI:** 10.3390/bs14070547

**Published:** 2024-06-28

**Authors:** Isabella Gural, Catia Cividini-Motta, Marissa L. Del Vecchio, Madeline R. Risse

**Affiliations:** Department of Child and Family Studies, University of South Florida, Tampa, FL 33620, USA; isabellagural6@gmail.com (I.G.); mdelvecchio@usf.edu (M.L.D.V.); mrisse@usf.edu (M.R.R.)

**Keywords:** ASD, self-monitoring, self-monitoring of performance, peer mediator

## Abstract

Self-monitoring (SM) is a widely used intervention to address a myriad of problem behaviors exhibited by individuals with autism spectrum disorder (ASD) and other disabilities (e.g., specific learning disability). SM of performance (SMP) interventions have been effective in increasing task completion and on-task behaviors in the general education setting. However, most of the research on SM interventions has been completed in a school setting, and few have evaluated the inclusion of a peer mediator component within a SM treatment package. Therefore, the purpose of this study was to evaluate the effects of a SMP intervention on disruptive behavior and task completion in three children with ASD. This study extends previous research by incorporating a peer mediator component, including children with ASD, and implementing the intervention in a clinic setting. The results show that the SM treatment package was effective, as the level of disruptive behavior and task completion improved for all three participants compared to baseline levels.

## 1. Introduction 

Disruptive (i.e., challenging) behaviors exhibited by children have been associated with poor short- and long-term outcomes, such as peer rejection [1], suspension [2], reduced academic performance [3], criminal activity, adult incarceration [4], placement in exclusionary settings, poor social development, and decreased social interactions [5]. Disruptive behaviors, which have been generally defined as any repeated pattern of behavior that inhibits learning or engagement with others [6], can take the form of a variety of topographies (e.g., self-injury, aggression, noncompliance; [2,7]). Disruptive behavior is commonly exhibited by neurotypical children and children with learning disabilities, emotional and behavioral disorders (EBD), attention-deficit/hyperactivity disorder (ADHD), and/or autism spectrum disorder (ASD; [3]) before 5 years of age [8]. Given that the presence of disruptive behavior exhibited in childhood has been linked with a host of negative outcomes that can persist into adulthood [8], intervention to reduce disruptive behavior is vital [3].

Due to the nature of disruptive behaviors, and the potential for these behaviors to negatively impact the learning of others in the educational environment [9], teachers have indicated that they allocate a significant amount of time to address these behaviors [7], which can ultimately lead to decreased academic performance (e.g., low grades, poor performance on standardized tests; [10]). As a result, teachers may be more inclined to utilize punishment-based procedures or reactive approaches [7], such as reprimands or classroom exclusion [11]. However, these approaches are ineffective in producing desired behavior change [12], increase children’s risk of social isolation, and reduce their ability to maintain interpersonal relationships [5]. Therefore, research suggests that it is critical to address disruptive behaviors as early as possible (i.e., before first grade; [7]) and to employ evidence-based interventions focusing on reinforcement instead of punishment-based procedures [13].

Theoretically, when a child’s level of disruptive behavior is low, there is a greater likelihood that academic engagement will increase, which has ultimately led to enhanced academic success [14]. One example of well-supported interventions for disruptive behavior exhibited by individuals with ASD is self-management [15], which entails teaching the learner to monitor, record, report, and reinforce their own behavior [16]. More specifically, self-monitoring (SM) is one of the most commonly used types of self-management interventions, and previous studies have evaluated SM in a variety of settings (e.g., hospitals, clinics, family homes, and school systems) with preschool and school-aged children with ASD [15,17]. Following a review of 66 studies, Bruhn et al. [3] concluded that SM is a highly effective intervention that produces drastic decreases in disruptive behavior and increases in academic engagement.

SM is an antecedent-based strategy used to teach individuals to identify and record the occurrence or nonoccurrence of one’s behavior [3]. SM is usually implemented as a multicomponent package that may include goal setting, peer or adult feedback, and/or contingent reinforcement [3]. SM is associated with immediate and impactful behavior change, is easily adaptable [3], promotes the generalization and maintenance of self-regulation skills [18], and is a relatively unobtrusive intervention within the natural environment [19]. Moreover, previous research has divided SM interventions into two categories: SM of attention (SMA) and SM of performance (SMP), and the findings of previous research indicate that both are equally effective [20]. The purpose of SMA interventions is to increase children’s awareness of a specific behavior so they can attend to an academic task [20,21]. SMA is best suited for children who have the skills necessary to complete academic tasks but engage in disruptive behaviors that negatively impact their level of attention to that task [20]. Conversely, SMP interventions aim to increase children’s level of task completion, thereby increasing the accuracy of the task and improving behavior [20,21]. SMP is recommended for children who have difficulty completing complex tasks, causing disruptive behavior to occur [20].

Rosenbloom et al. [22] demonstrated the effects of a SMA intervention that utilized the I-Connect application to increase on-task behavior and decrease disruptive behavior in an elementary-aged child with ASD. Risse et al. [23] used a SMP intervention, which utilized the free List application, with differential reinforcement to decrease off-task behavior and increase task completion of three fifth grade students with ADHD, specific learning disabilities (SLD), and/or speech and language impairments (LI) in the general education setting. In this study, throughout the typical academic period, students earned points based on their percentage of task completion and accuracy of SM and exchanged the points for backup reinforcers. These findings are promising given that the teacher did not have to alter existing classroom management strategies to target off-task behavior other than implementing the SMP intervention. Holifield et al. [24] reported that a SMP intervention effectively increased both attending to the tasks and the accuracy of task completion in two elementary-aged children diagnosed with ASD compared to baseline levels. Rosenbloom et al. [25] used a technology-based SM application to target both SMA and SMP for four adolescents with ASD, and in this study, the SM intervention led to increases in on-task behavior and task completion for all four participants.

Few studies have evaluated SM interventions that include an adult- or peer-monitoring component. For example, Shearer et al. [26] evaluated the differential effects of an SM package with an adult-mediator and peer-mediator component to increase social skills exhibited by three children with ASD. They found that the intervention package was successful in producing increases in social engagement for all three participants, with no differences between the adult and peer mediators. Additionally, Carr et al. [27] observed increases in children’s social skills when self-management interventions included a peer mediator component. However, only 17% (*n* = 4) of the 23 studies included in the review involved a form of peer component within a SM package [27]. Morrison et al. [28] aimed to increase social skills exhibited by four students with ASD using a SM package that included a peer mediator and peer-monitoring component. The peers involved in this study were the same age as the participants but did not have a diagnosis of ASD. The results showed that the SM package was effective in teaching social skills to all four participants while simultaneously decreasing each participant’s level of inappropriate behaviors [28]. Roberts et al. [29] implemented a SM intervention package with a peer-trainer component to increase academic engagement for two high school students with ASD. The authors reported that the peer trainer, who was also diagnosed with ASD, implemented the intervention with fidelity, and moderate increases in academic engagement for both participants were observed. Finally, following a review of the literature, Lee et al. [17] suggested that SM interventions that involve an adult- or peer-monitoring component may be more effective than SM interventions that involve the participant alone. Moreover, the inclusion of a peer mediator is not only a more contextually fit approach but may increase the effectiveness and likelihood of generalization compared to adult-mediated interventions [30,31].

Although the specific mechanisms related to the increased effectiveness of peer-mediated relative to non-peer-mediated interventions are unclear, previous research has offered plausible explanations. First, peer-mediated interventions lead to increased opportunities for participants to engage in the target skill within a variety of contexts and with multiple peers, resulting in contact with naturally maintaining contingencies that may not be otherwise contacted through adult-mediated interventions [30]. According to Chan et al. [32], the inclusion of peer-mediated interventions may foster inclusion in school settings, resulting in an increased level of contingent social reinforcement when the participant engages in the target skill. Additionally, given that the number of peers is exceptionally larger than the number of adults in school and community settings, the incorporation of a peer mediator will result in an increased number of intervention agents. With this, the expectations and demands on adults will be lessened, which will simultaneously promote increased intervention implementation for the participant [32], and potentially lead to enhanced treatment outcomes.

Previous research has reported the successful implementation of SM interventions within a variety of settings; however, the outcomes of several literature reviews indicate that they are used most often in schools and educational settings [15,17,27]. Specifically, Carr [15] reported that only three studies out of the 12 that were included in their review were implemented within the clinical setting (i.e., in a clinic providing behavioral services), and all of these were published prior to the year 2000. Koegel et al. [33] implemented a self-management intervention that included a SM component to increase social skills for four children with ASD across clinic and community settings. The results indicated that the self-management intervention with a SM component was responsible for increased appropriate responses and decreased disruptive behavior across clinic and community settings for all four participants [33]. Stahmer and Schreibman [34] used a self-management treatment package that included SM to increase appropriate play skills and decrease inappropriate and self-stimulatory behavior for three children with ASD in a clinic and home setting. The authors also reported that the level of appropriate play, inappropriate behavior, and self-stimulatory behavior generalized to novel settings [34]. Pierce and Schriebman [35] used a self-management package with picture prompts and SM to teach daily living skills to three boys with ASD in a clinic setting. They reported that the intervention led to a significant and immediate increase in the percentage of on-task behavior and a substantial decrease in the level of inappropriate behavior for all three participants compared to baseline levels. Moreover, when generalization and 2-month follow-up maintenance probes were completed, the level of these behaviors remained similar to those attained during the intervention [35]. The results from these studies show that SM intervention packages implemented in a clinic have produced meaningful changes in a variety of behavior topographies and have successfully generalized to novel settings and/or maintained in follow-up phases.

Evidentially, SM interventions have been effective across a range of behaviors (e.g., [23]), settings (e.g., [24,35]), and with the inclusion of additional treatment components (e.g., peer mediators; [29]) for youth diagnosed with ASD. Despite advances in the literature, there is still a need to evaluate the effectiveness of a SM treatment package similar to that described by Risse et al. [23] with the addition of a peer mediator implemented in a clinic for children with ASD. Previous research suggests that the incorporation of a peer mediator is especially effective in increasing the social skills of children with ASD [30], which has been associated with a decrease in disruptive behavior (e.g., [33]). Furthermore, when disruptive behavior is decreased, academic engagement and overall academic success is likely to increase [14]. Therefore, it is possible that a reciprocal relationship between social skills, academic engagement (e.g., task completion), and disruptive behavior might exist. Thus, the purpose of this study was to replicate the study completed by Risse et al. [23] by evaluating the effects of a technology-based SM intervention on task completion and disruptive behavior. This study extended the procedures employed by Risse et al. [23] by including a different population (i.e., children with ASD), a peer mediator, and a novel setting (i.e., a clinic instead of a regular classroom).

## 2. Method

### 2.1. Participants and Setting

This study included three child participants, five clinicians (i.e., registered behavior technicians; RBTs), and two peer mediators. We are reporting their race or ethnicity based on the information each participant or caregiver provided. Prior to the onset of data collection, we obtained caregiver consent for the child participants and the peer mediators to participate in the study. Moreover, we obtained formal assent from the child participants and peer mediators and consent from all participating RBTs.

We conducted sessions two to three times per week at a therapy room in an applied behavior analysis (ABA) clinic, and sessions lasted 10–20 min. The therapy rooms included tables, chairs, toys, and various miscellaneous items (e.g., markers, sensory toys) for use during clinic sessions, and usually the only individuals present in the room were the participating RBT, peer mediator, and child participant. We completed the sessions during the child participants’ regularly scheduled therapy sessions, during times when the RBTs presented academic tasks (e.g., math worksheets) to the child participants and instructed them to complete these independently (i.e., “1:1 table work”). The RBTs identified these as the times associated with the highest level of disruptive behaviors.

#### 2.1.1. Child Participants

Child participants were 6-, 8-, and 9-year-old children who displayed high levels of disruptive behaviors (i.e., 30% or more of the intervals of the initial observation session) and low rates of task completion (i.e., fewer than 50% of assigned tasks completed during initial observation). Austin was a 6-year-old White boy diagnosed with ASD, ADHD, and oppositional defiance disorder. Austin communicated vocally using full sentences, and at the time of his participation in this study, he was receiving 15 h per week of clinic-based ABA services. Kyle was an 8-year-old Hispanic boy diagnosed with ASD. Kyle communicated vocally in English and Spanish using short phrases (e.g., “I want iPad” or “I want more time”). Kyle was receiving 25 h per week of clinic-based ABA services. Juliet was a 9-year-old White girl diagnosed with ASD and ADHD. Juliet communicated vocally in full sentences and was receiving 25 h per week of clinic-based ABA services.

#### 2.1.2. Peer Mediators

The RBTs nominated the peer mediators. Peer mediators were children that received ABA services at the same time as the participating child but had no strong (positive or negative) learning history with the child participant. Peer mediators engaged in minimal disruptive behaviors (i.e., 10% or fewer intervals of the initial observation session) and completed tasks as assigned during sessions. Ricky was a 10-year-old White boy diagnosed with ASD who communicated vocally using full sentences and was receiving 10 h per week of clinic-based ABA services. He was the peer mediator for Austin and Juliet. Joe was an 8-year-old White boy diagnosed with ASD who communicated vocally using short phrases (e.g., “It’s time to do work” or “Play with me”) and was receiving 25 h per week of clinic-based ABA services. He was the peer mediator for Kyle.

#### 2.1.3. RBTs

Six RBTs—Erica, Lauren, Lilian, Summer, Emma, and Wendy—participated in the study. The inclusionary criterion for the RBTs consisted of willingness to implement the intervention. Austin’s RBTs were Erica, Lauren, Lillian, and Summer. Kyle’s RBTs were Summer and Emma. Juliet’s RBTs were Summer, Lauren, and Wendy. Erica was a 25-year-old Caucasian female. Lauren was a 28-year-old Hispanic female. Lillian was a 60-year-old Caucasian female. Summer was a 23-year-old White female. Emma was a 21-year-old Hispanic female. Wendy was a 21-year-old Hispanic female. Erica, Lauren, and Lilian had 2 years of experience, Summer had 1 year of experience, and Emma and Wendy had 2 months of experience working as an RBT.

### 2.2. Materials

Materials used in this study included data sheets, a pen, and a timer for data collection. Session stimuli included a tablet with the Kids ToDo List^®^ application (Mac OS v.1.0.7), specified reinforcers for the target participant and peer mediator, and task-related materials. The independent work tasks consisted of worksheets (e.g., math, tracing, matching worksheets), matching activities, sorting tasks, and puzzles for Austin and Kyle. Juliet’s tasks included reading and math worksheets of varying difficulty. The Kids ToDo List^®^ application was chosen because it is a free application that is available for both Apple and Android products, it is user-friendly (e.g., it requires few steps to set up), and task lists can be individualized to the learner (e.g., multi-word label of tasks, single word label, or word and picture labels). The application also contains a visual stimulus showing the user’s progress (i.e., a star next to items that have been completed with a progress bar showing the percentage of completion).

### 2.3. Dependent Variables and Measurement

The primary investigator (P.I.) and a trained research assistant (RA) collected data for the study on three dependent measures: disruptive behavior, task completion, and points earned. The P.I. and RA were both graduate students at the University of South Florida.

#### 2.3.1. Disruptive Behavior

We developed individualized definitions for the disruptive behavior of each child participant based on input from the RBTs. Due to the nature of the assigned tasks (i.e., independent work), for all three participants, any vocalizations (e.g., singing, humming, talking) other than requesting assistance with the task (all three participants) or asking how much time was left in the work session (Juliet) were considered disruptive behavior. For Austin, disruptive behavior also included any instance of property destruction that resulted in audible sound (i.e., hitting a pencil on the table, throwing a toy on the floor, or hitting the table) and any instance of being disengaged from the task for 5 s or longer (i.e., looking out the window, putting his head down on the table, or looking around the room). For Kyle, disruptive behavior also included property destruction (i.e., ripping his worksheet or throwing work material), physical aggression towards others (i.e., hitting others with an open hand, biting, kicking, or throwing objects at others), self-injurious behavior (i.e., biting his hand), or being disengaged from the task for 5 s or longer (i.e., playing with other items in the room). For Juliet, disruptive behavior also included property destruction (i.e., kicking the wall), physical aggression towards herself or others, or being disengaged from the task for 5 s or longer (i.e., looking around the room, engaging in a task other than the instructed task, or walking around the room). We measured disruptive behavior using a 10-s partial interval recording system. To calculate the percentage of intervals with disruptive behavior for each session, we divided the number of intervals with disruptive behavior by the total number of intervals in the session and multiplied by 100.

#### 2.3.2. Task Completion

Task completion consisted of the child participant completing the task assigned to them within a specified time (e.g., providing answers to all math problems in the math worksheet provided by the RBT), independently of whether the child participant completed the task with or without assistance and/or additional clarification of task expectations from the RBT. Examples include providing an answer to all math problems on a worksheet, tracing the sentences on a tracing worksheet, and locating all words for a word search activity, irrespective of accuracy. During each session, the RBT presented three to five tasks to the child participant, and after the session ended, the P.I. and RA recorded data on the number of tasks completed by reviewing the permanent products. The P.I. divided the number of completed tasks by the total number of tasks that were presented during the session and multiplied by 100 to yield a total percentage of task completion for each session.

#### 2.3.3. Points Earned

Points earned refer to the number of points awarded to the child participant during each session of the intervention phase. The RBT assigned points for task completion and accuracy of SM (see differential reinforcement section for additional information). Therefore, the total possible points per session varied based on the number of tasks assigned for that session. The P.I. summarized these data by dividing the number of points earned by the total possible points and multiplying by 100 to yield the total percentage of points earned per session.

### 2.4. Interobserver Agreement

To calculate interobserver agreement (IOA), an RA independently collected data on each of the dependent measures using video recordings of sessions for a minimum of 33% of sessions across phases for each child participant. The P.I. utilized behavioral skills training (BST; [36]) to train the RA to proficiency on the operational definitions and corresponding measurement strategies for each dependent measure before the RA independently collected data for assessments of IOA. The P.I. and RA were required to attain an IOA score of 90% or higher for at least one practice session before beginning data collection independently for the study. See Table 1 for detailed IOA scores.

We calculated IOA scores for disruptive behavior using the interval-by-interval method, which entailed dividing the number of intervals with agreement between the data collected by the two observers (i.e., P.I. and RA) by the total number of intervals in the session. We calculated IOA scores for task completion using the item-by-item method by dividing the number of tasks with agreement (i.e., scored as completed) between observers by the total number of tasks presented during that session, multiplied by 100. For points earned, we calculated total agreement IOA by comparing the number of points earned recorded by the P.I. and the RA, dividing the smaller number of reported points earned by the largest number of reported points earned, and then multiplying by 100.

We calculated IOA scores for 33% of baseline and 43% of intervention sessions for Austin. The mean IOA score for Austin’s disruptive behavior was 83% (range: 65–93%). We calculated IOA for 40% of baseline and 33% of intervention sessions for Kyle. The mean IOA for Kyle’s disruptive behavior was 86% (range: 71–100%). Finally, we calculated IOA for 33% of baseline and 50% of intervention sessions for Julia. The mean IOA for Julia’s disruptive behavior was 90% (range: 80–97%). The mean IOA for task completion and for points earned was 100% across baseline and intervention phases for Austin, Kyle, and Julia.

### 2.5. Procedural Fidelity

To determine the accuracy of the implementation of intervention sessions by the RBT and the peer mediator, the P.I. used two checklists, one consisted of a task analysis of the steps the RBT and the other of the steps the peer mediator should implement during the SMP intervention. The P.I. recorded procedural fidelity data using the checklists for 57% of intervention sessions for Kyle and 50% of intervention sessions for Austin and Juliet. Procedural fidelity was calculated by dividing the number of steps completed correctly by the total number of steps in the checklist and multiplying by 100. The mean procedural fidelity score across RBTs was 94% (range: 82–100%). The mean procedural fidelity score for the peer mediator Ricky was 81% (range: 50–100%) when he was assisting Austin and 87.5% (range: 75–100%) when he was assisting Juliet; the mean procedural fidelity score was 83% for Joe (range: 75–100%).

We also assessed, using a training fidelity checklist, the implementation fidelity of the training procedures used during RBT-implemented training conducted with the child participant and peer mediator. The implementation fidelity of training was calculated by dividing the number of steps completed correctly by the total number of steps in the checklist and multiplying by 100. The mean implementation fidelity of RBT-delivered training was 100% across all training sessions. See Table 2 for a detailed description of procedural fidelity results across participants and phases.

### 2.6. Social Validity

After the conclusion of the intervention phase, we collected social validity data from the child participants, RBTs, and peer mediators using a researcher-developed questionnaire. All questionnaire responses included a 5-point Likert rating scale, with 1 = strongly disagree and 5 = strongly agree. The child participants completed a 5-item questionnaire, RBTs completed a 6-item questionnaire, and peer mediators completed a 4-item questionnaire evaluating their acceptability of the intervention goals, procedures, and outcomes. The results of the social validity assessments can be found in Table 3 (child participants), Table 4 (RBTs), and Table 5 (peer mediators).

### 2.7. Experimental Design

This study utilized a nonconcurrent multiple-baseline-across-participant design [37]. All participants experienced the baseline condition until visual inspection of the data indicated responding was stable or consisted of a countertherapeutic trend. We stagged the introduction of the SM intervention across participants and the intervention was introduced only after an effect of the intervention was seen for the previous participant and the data for the participant showed a stable or countertherapeutic trend.

### 2.8. Procedures

#### 2.8.1. Pre-Experimental Assessments

**Initial Observation.** The P.I. conducted a 10–20 min observation to determine if the child participants and peer mediators met inclusionary criteria. Austin’s observation lasted 10 min, and he engaged in disruptive behavior during 56% of the intervals of the observation and completed 0% of the tasks presented. Kyle’s observation lasted 11 min, and he engaged in disruptive behavior during 92% of the intervals of the observation and completed 33% of the tasks presented. Finally, Juliet’s observation was 15 min, and she engaged in disruptive behavior during 39% of the intervals and completed 25% of the tasks presented. The peer mediators, Ricky and Joe, were observed for 10 min and 20 min, respectively. Ricky engaged in disruptive behavior during 6% of the intervals of the observation and completed 100% of the tasks presented, while Joe engaged in disruptive behavior during 10% of the intervals of the observation and completed 75% of the tasks presented.

**Preference Assessment.** After the initial observation, the P.I. completed an indirect preference assessment using procedures like those described by Northup [38] with each child participant and peer mediator using stimuli the RBTs identified as potential reinforcers for each child participant and peer mediator. The P.I. created a list of available items and provided it to the child participant and peer mediator to rank the items from most preferred to least preferred. The P.I. then created a reinforcer menu with the identified items, and each item was assigned a point value based on their level of preference. Austin’s ranked reinforcers, from highest preferred to least preferred, were free time with toys, tablet videos, and playing in the occupational therapy room. Kyle’s ranked reinforcers, from highest to lowest preferred, were the tablet, the occupational therapy room, and the playground. Juliet’s ranked reinforcers, from highest to lowest preferred, were talking about Pokémon with adults, swinging, and playing a board game.

#### 2.8.2. SMP Evaluation

**Baseline.** Baseline sessions lasted up to 20 min in duration, depending on the targeted task. During the baseline phase, the RBT implemented the typical clinic session procedures, and the P.I. instructed them to respond to disruptive behavior as usual. During baseline, the RBTs presented three to five tasks (e.g., worksheets, matching tasks) and set a timer up to 20 min depending on the number of tasks presented. The Kids ToDo List^®^ was not used during these sessions, as no components of the intervention were in place during baseline. The P.I. recorded data on disruptive behavior and task completion for each session.

During baseline, the consequences for appropriate and disruptive behavior of the child participants varied, as these were based on the programming in effect for each child participant. All RBTs responded to requests for assistance and/or Juliet’s inquiries regarding how much time was left in the session. For Austin’s sessions, the RBT did not provide any consequences for disruptive behavior after reviewing the assigned tasks. The RBT’s consequences for Kyle’s disruptive behavior included providing no consequences (i.e., ignoring it) and occasionally verbally redirecting him to his assigned tasks. Juliet’s RBT presented the task instructions and then provided verbal attention and redirection in response to all topographies of disruptive behavior, except for physical aggression, in which the RBT implemented physical response blocking.

**Intervention Training for the RBT.** Before the SM intervention, the P.I. conducted two training sessions with the participating RBTs prior to the scheduled clinic sessions with the child participants. The sessions lasted approximately 15 min. The P.I. implemented BST, beginning by reviewing the steps to implement the intervention, modeling each step, allowing the RBTs to rehearse/roleplay the steps, and providing feedback to the RBTs. The first training session focused on the steps the RBT needed to complete during implementation of the intervention, whereas the second session focused on the steps the RBT had to implement when providing training to the target student and peer mediator. After the two training sessions, all RBTs implemented the procedures with at least 90% fidelity across two role-play scenarios and reported that they were prepared to implement intervention.

**Child Participant and Peer Mediator Training**. Following baseline, the RBTs utilized BST to teach the child participant how to use the SM intervention using Kids ToDo List^®^ and train the peer mediator how to assist with implementation of the procedures (e.g., review the list of tasks, help collect materials, deliver earned reinforcer). Training continued until the target student and peer mediator demonstrated 90% or higher correct implementation of the SM intervention during two role play scenarios. All participants and peer mediators required only one training session, lasting approximately 15 min to meet the mastery criteria.

**SMP.** During the SM phase, child participants used the Kids ToDo List^®^ application on a tablet to track the tasks they had completed during a session. Prior to beginning the session, the RBT provided the P.I. with the assignments they were going to present to the child participant for each session, and the P.I. input each required assignment into the Kids ToDo List^®^. For all participants, the list of assigned tasks consisted of names and pictures of the tasks. The peer mediator reviewed the list of assignments with the child participant at the onset of the session, and the RBT ensured all materials were available and answered questions regarding the assignments. Throughout the session, the child participant had continuous access to the tablet with the Kids ToDo List^®^ application, which specified the tasks assigned for that session, and usually the child participant placed the tablet next to themselves. As the child participant completed each task, they checked the box next to the name of the corresponding assignment using the Kids ToDo List^®^ application. Subsequent to checking off the task they had completed, the child participant reviewed (e.g., pointed to or read) the next item in the list, gathered the related materials, and worked on the next task. This process was repeated until the child participant completed all tasks on the list. The peer mediator remained near the child participant, working on the tasks assigned to them, but the peer did not prompt the child participant to complete the tasks.

Upon completion of all items in the list, which signaled the conclusion of the session, the child participant gathered all materials and turned them into the RBT (complete and incomplete tasks). The RBT checked the work products to determine which tasks were complete and the corresponding number of points earned. The RBT directed the peer mediator to gather the specified reinforcer earned by the child participant and by the peer mediator, and the peer mediator delivered the reinforcer to the child participant and to themselves. The intervention sessions lasted up to 20 min, depending on the tasks assigned by the RBT to the participant. Moreover, like in baseline, during each session the RBT assigned 3 to 5 tasks (e.g., 3 worksheets; 1 worksheet with 3 math problems) to the participant, and set a timer for 10–20 min, depending on how much time the RBT deemed necessary for the participant to complete the task.

***Differential Reinforcement.*** After each session, reinforcers were provided to the child participant based on the number of points earned and to the peer mediator based on the assistance they provided. The RBT assigned points to the child participant based on the number of tasks completed and the accuracy of their SM of task completion in comparison to the RBT’s report of task completion (via a checklist created by the P.I. of the assigned tasks). The RBT assigned two points for each task that both the child participant and the RBT marked as completed, and the child participant completed the task (i.e., the child participant completed the task and their data matched). The RBT assigned one point to each task the participant did not complete, and both the child participant and the RBT marked as not complete (i.e., the child did not complete the task, but their data matched). When the data recorded by the child participant and RBT did not match (e.g., the RBT recorded that the child did not complete the task whereas the child indicated they completed the task), the RBT assigned 0 points to that task. This system is based on the system implemented by Peterson and colleagues [39], which compared the data recorded by a teacher to the data recorded by the student.

The criteria for the child participants to receive their highest preferred reinforcers were determined based on their baseline level of disruptive behavior and task completion. The criteria to access their highest preferred reinforcer for Austin and Kyle consisted of earning at least 75% of the possible points, whereas for Juliet it consisted of earning 100% of the points. The criteria to access a moderately preferred reinforcer for Austin and Kyle consisted of earning 50% to 74% of the possible points, and for Juliet, earning 75% to 99% of the possible points. The criteria to access the lowest preferred reinforcer consisted of earning 25% to 49% of the possible points for Austin and Kyle and earning 50% to 74% of the possible points for Juliet. The criteria to receive a reinforcer for the peer mediator were based on whether they assisted (e.g., reviewed the list of tasks with the child participant, helped them collect materials, delivered the earned reinforcer) with the intervention. The peer mediator received their highest preferred reinforcer if they assisted the child participant and the RBT; no reinforcer was provided if they did not assist at all during the intervention. Both peer mediators received their highest preferred reinforcer for all intervention sessions.

## 3. Results

**Figure 1:**
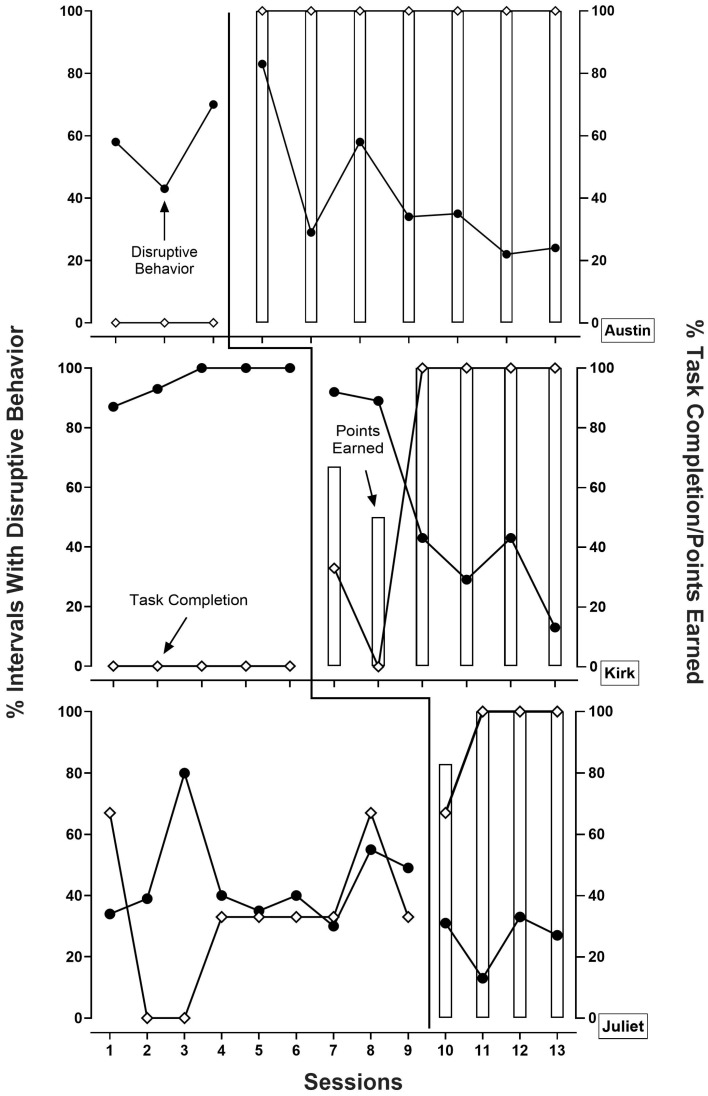
Percentage of 10-s intervals with disruptive behavior, percentage of tasks completed per session, and percentage of points earned per session across all phases and participants.

Figure 1 depicts the percentage of intervals with disruptive behavior, the percentage of tasks completed, and the percentage of points earned across baseline and SM phases for all child participants. During baseline, Austin did not complete any of the tasks (i.e., completed 0% of the tasks) and engaged in disruptive behavior during an average of 57% (range: 43–70%) of the intervals of each session. During the intervention sessions, Austin completed 100% of the tasks presented during each intervention session, and disruptive behavior decreased to an average of 41% of intervals in each session (range: 22–83%). Across all the intervention sessions, Austin earned 100% of the possible points.

During baseline, Kyle did not complete any of the tasks (i.e., completed 0% of the tasks) and engaged in disruptive behavior during an average of 96% (range: 87–100%) of the intervals of each session. During intervention, Kyle completed an average of 72% (range: 0–100%) of tasks presented during each session, and disruptive behavior decreased to an average of 52% (range: 13–92%) of the intervals of each session. Across the intervention sessions, Kyle earned an average of 86% (range: 50–100%) of the possible points.

Finally, during baseline, Juliet completed an average of 33% (range: 0–67%) of the tasks per session and engaged in disruptive behavior during an average of 45% (range: 30–80%) of the intervals of each session. During the intervention, Juliet completed an average of 92% (range: 67–100%) of tasks during each intervention session, disruptive behavior decreased to an average of 26% (range: 13–33%) of the intervals of each session, and Juliet earned an average of 96% (range: 83–100%) of the possible points. Due to Juliet transitioning to another service provider, we only completed four intervention sessions with her. 

Table 3 (child participants), Table 4 (RBTs), and Table 5 (peer mediators) contain the results of the social validity assessments. For the child participants, the mean social validity scores were 5 for Austin, 4.2 (range: 3–5) for Kyle, and 4.4 (range: 3–5) for Juliet. Austin reported that his favorite part of participating in the intervention was being able to earn more free time. Kyle reported that his favorite part of participating in the intervention was earning time with his tablet. Juliet reported that her favorite part of participating in the intervention was getting to work with her friend (i.e., receiving assistance from the peer mediator). For the peer mediators, Ricky’s mean social validity score was a 5 and Joe’s was a 4 (range: 3–5). For the RBTs, Erica’s mean social validity score was 4.8 (range: 4–5), Summer’s 4.7 (range: 3–5), Lauren’s 4.7 (range: 4–5), and Lillian’s 4.2 (range: 3–5). We did not assess the social validity of Wendy, as she only participated in baseline sessions. The results of the social validity assessments indicate unanimous satisfaction with the intervention procedures, as inferred by the responses indicating the intervention was easy (RBT, peer, and child participant), did not drastically reduce class time (RBT), nor impede completion of their own work (peer), agreement that the intervention was effective, as inferred by the RBTs response indicating the intervention helped their “student(s) stay focused on their tasks”, and desire to participate in (peer and child participant) or implement the intervention in the future (RBT).

## 4. Discussion

This study evaluated the effects of an SM intervention with the inclusion of a peer mediator on the disruptive behavior and task completion of three children with ASD (i.e., the child participants) within a clinic setting. The results indicate that, following implementation of the intervention, disruptive behavior for all three child participants decreased from baseline levels and demonstrate a functional relation between the intervention and increased task completion for all three child participants. However, it is important to note that the number of intervention sessions required for the intervention to result in a substantial change in levels of disruptive behavior and task completion varied across participants. More specifically, the onset of the intervention led to an immediate decrease in disruptive behavior and task completion for Juliet; for Kyle, disruptive behavior decreased only slightly during the first couple intervention sessions, and task completion increased slightly during the first session, then decreased, and then increased to 100% during the 3rd session and remained at 100% for all subsequent sessions. During baseline, the levels of disruptive behavior and task completion differed across participants, and this variability in baseline may have contributed to the differing number of intervention sessions needed for the intervention to affect both disruptive behavior and task completion. Additionally, RBTs reported that the SM intervention, with the inclusion of a peer mediator, helped the child participants attend to tasks and indicated that they were likely to use this intervention again in the future. Peer mediators and child participants indicated that their role in the SM intervention was “easy.” Peer mediators stated that they would participate in the intervention again in the future, and child participants believed that their peers may enjoy participating in this intervention as well. The results from these assessments suggest that the SM intervention with a peer mediator component has strong social validity across RBTs, peer mediators, and child participants. Additionally, procedural fidelity remained above 80% for all peer mediators and RBTs, with most (i.e., five of six) procedural integrity scores above 90% for RBTs.

This study supports and adds to the SM literature in three notable ways. First, the procedures of the current study differed from those employed by Risse et al. [23] by including different participants (i.e., children with autism; RBT instead of a teacher), conducting sessions in a different setting (i.e., an ABA clinic), and adding a peer component. Regarding the setting, in the current study only the participants (RBT, peer, child participant) were present in the room, whereas the room (i.e., classroom) used by Risse et al. included a teacher and up to 25 students; in the current study, the RBT presented academic tasks to the child participant, whereas in the Risse et al. study, academic tasks were presented by the teacher; and in the Risse et al. study, the same academic tasks were presented by the teacher to the entire group of students, whereas in the current study, the RBT presented different academic tasks, based on each child’s educational plan and chosen by the RBT, to the peer and the child participant. Second, according to the review completed by Carr [15], this is one of only a few studies that evaluated the effectiveness of SM outside of a school setting; the three studies identified in the review, which were conducted in a clinic, were published before the year 2000 and did not include a peer component [15]. Furthermore, although disruptive behavior was the dependent variable for two of the three studies (i.e., [33,35]), these studies did not evaluate the effects of a SM intervention on task completion. Third, previous research evaluating peer-mediated interventions has rarely targeted the same dependent variables as those included in the current study. In many cases, peer-mediated SM interventions have been used to increase the social skills of children with ASD (e.g., [28,30,33]). One exception is the study completed by Canfield and Cividini-Motta [40], which evaluated the effects of a peer-mediated daily behavior report card intervention on the disruptive and on-task behavior of school-aged children identified as at risk for receiving an EBD classification.

SM has strong contextual fit due to the feasibility [19] and adaptability [3] of the intervention. For example, Risse et al. [23] noted that the classroom teacher did not need to modify any preexisting classroom management strategy upon the implementation of the SMP intervention. Yet, the contextual fit of SM can be further enhanced when components such as the inclusion of a peer mediator [30] and the intervention modality (i.e., paper/pencil- vs. technology-based SM; [41]) are modified. Previous research suggests that SM with the inclusion of a mediator is more effective than SM with the participant alone [17]; but the inclusion of a peer compared to an adult mediator is more advantageous in promoting desired behaviors for children with ASD and has stronger contextual fit [30]. Additionally, Bouck et al. [41] reported that the participants’ level of task independence was highest in the technology-based SM modality compared to paper/pencil SM. Thus, the current SM intervention is unique as the procedures that were employed have incorporated both technology-based SM (i.e., the Kids ToDo List^®^) and peer mediators, resulting in stronger contextual fit compared to interventions that have one or none of these components.

The SM intervention evaluated in this study consisted of multiple components, and it is plausible that one component in isolation or the combination of multiple components was responsible for the treatment effects observed. Therefore, it is important to consider the potential mechanisms responsible for the treatment effects obtained. First, the Kids ToDo List^®^ included written and pictorial information. These stimuli likely served as prompts and occasioned the initiation of each task. Related to this, given that the application remained available throughout the session, these prompts were also continuously available. Thus, it is possible that task completion increased because prompts were continuously available in comparison to the vocal prompts presented by the RBT during baseline, which are transitive in nature. Although prompts occasion responses, the probability of a response occurring again in the future is dependent on whether it encounters reinforcing or punishing consequences. Given that the SM intervention included a differential reinforcement component, which consisted of providing the highest preferred item contingent upon higher task completion, and reinforcing consequences increase the probability of the response occurring again in the future, it is likely the differential reinforcement component was responsible, at least in part, for the outcomes of the SM intervention. Likewise, assuming that interactions with the peer were reinforcing, which is supported by the fact that Juliet indicated she enjoyed working with her peer, it is plausible that the involvement of the peer could have increased task completion by decreasing the aversiveness of completion (e.g., pairing of a neutral or aversive stimulus with a reinforcing stimulus) or by serving as a reinforcer for task completion. To clarify, given that peer mediators delivered the preferred stimulus to the child participant at the end of the session, task completion resulted in access to a preferred stimulus and peer attention.

Despite the effectiveness of this intervention, this study is not without its limitations. First, we completed this study in an ABA clinic, and the number of other individuals (e.g., other RBTs, children) present in the room during sessions varied but never exceeded more than three other children and their respective RBTs. It is unclear if equivalent results would be attained if the intervention was implemented in a classroom within a school setting, as the number of children within a classroom would vary greatly compared to the small groups that were present in this study. Second, for Juliet, the intervention ceased before a substantial decrease in disruptive behavior was obtained because she moved to another service provider. However, despite a small number of intervention sessions for Juliet, an immediate decrease in disruptive behavior and an immediate increase in task completion were observed upon the implementation of intervention. It is reasonable to assume that if intervention continued, similar levels of response or further decreases in disruptive behavior would have been observed. Third, this study evaluated an intervention consisting of multiple components (e.g., self-monitoring application; reinforcement); thus, it is unclear which components of the intervention were responsive for the treatment outcomes. Future research should conduct a component analysis of the various components of this intervention to determine which are necessary. Another limitation of the study is the inclusion of multiple RBTs for each of the participants. Unfortunately, due to system-wide changes within the clinic, RBT turnover rate was high, which led to the recruitment of new RBTs for participation throughout the course of this study. However, it is plausible that the inclusion of multiple RBTs during the study may facilitate generalization of the outcomes to novel RBTs. Additionally, we did not assess the accuracy of the tasks completed by the child participants. However, anecdotal reviews of the work products indicated that participants usually provided accurate responses to the assigned tasks. Furthermore, the number of tasks presented per session varied because it was dependent on the RBT. Despite this limitation, given that task completion for all participants substantially increased during the intervention, the results suggest that the inconsistent number of tasks did not impact the efficacy of the intervention. Moreover, the variable number of tasks across sessions may simulate contingencies within the natural environment and may be useful to promote generalization without the incorporation of a systematic method to progressively increase task demands. However, our study did not include measures of generalization and maintenance of treatment effects, which is an additional limitation of this study. Future studies should assess the generalization and maintenance of treatment outcomes and evaluate the level of the participants’ task accuracy in addition to task completion. Finally, the definitions of disruptive behavior, which were developed in consultation with the RBT, may be considered stringent (e.g., singing and talking were considered disruptive behaviors). However, the definitions we employed are similar to those used in other studies that evaluated the impact of self-monitoring interventions within school settings and which took the expectations of teachers into consideration (e.g., [25]). It is important to note that although the study was conducted in an ABA clinic, for all child participants and peers, a long-term goal was to transition them into a less restricted setting, such as a regular school. Nonetheless, we recommend that future researchers, clinicians (e.g., RBTs), and educators include the client and all relevant stakeholders in discussions about the need for intervention (e.g., whether singing warrants an intervention) and appropriate goals for each of their clients or students.

To conclude, the results of this study indicate that technology-based SM with the inclusion of peer mediators may be an acceptable, effective, and feasible intervention for practitioners aiming to decrease disruptive behaviors exhibited by children with ASD in a clinic setting. When academic engagement (i.e., task completion) increases, a plethora of negative outcomes (e.g., peer rejection, suspension, reduced academic performance; [1,2,3]) can potentially be avoided. The findings from this study support the notion that a reciprocal relation may exist between the decrease in disruptive behaviors and the increase in academic engagement. Furthermore, although the current intervention included a peer component, this component can fade once the learner can independently complete all steps (e.g., gather materials, use SM tools, gather their earned reinforcers) which means technology-based SM can increase the independence of the learner, which in turn gives the teacher additional time to support learners who need additional assistance with the assigned tasks.

## Figures and Tables

**Table 1:** Mean IOA scores for each child participant across dependent measures.

	Disruptive Behavior		Task Completion	
	Mean IOA BL *(% of Sessions)*	Mean IOA SMP *(% of Sessions)*	Mean IOA BL *(% of Sessions)*	Mean IOA SMP *(% of Sessions)*
Austin	81% *(33%)*	84% *(43%)*	100% *(67%)*	100% *(43%)*
Kyle	97% *(40%)*	79% *(33%)*	100% *(40%)*	100% *(33%)*
Juliet	90% *(33%)*	86% *(50%)*	100% *(33%)*	100% *(50%)*

Note: BL = baseline; SMP = self-monitoring of performance.

**Table 2:** Procedural fidelity scores for each of the RBTs and peer mediators.

Individual	RBT Training of Target Student/Peer Mediator	Mean (and Range) of PF Scores During/(% of Sessions with PF)
Ricky (peer mediator for Austin)	N/A	81% (50% to 100%)/ (57%)
Joe (peer mediator for Kyle)	N/A	83% (75% to 100%)/ (50%)
Ricky (peer mediator for Juliet)	N/A	87.5 (75% to 100%)/ (50%)
Erica (RBT for Austin)	100%	100%/ (82%)
Lauren (RBT for Austin)	N/A	100%/ (67%)
Lillian (RBT for Austin)	N/A	100%/ (100%)
Summer (RBT for Kyle)	100%	100% (50%)
Emma (RBT for Kyle)	N/A	91%/ 50%
Summer (RBT for Juliet)	100%	92% (92% to 92%)/ 50%

*Note:* PF = procedural fidelity; ranges are not provided in cases where procedural fidelity was assessed for only one session; N/A = not applicable.

**Table 3:** Social validity scores for the child participants.

Item	Austin	Kyle	Juliet
Using the tablet helped me do my work.	5	5	3
Using the tablet was easy.	5	5	5
I liked using the tablet while completing my work.	5	3	4
I would like to use the tablet again in the future.	5	3	5
I think my friend would like to use the tablet as well.	5	5	5
Mean Scores	5	4.2	4.4
What did you like most—working with your friend, receiving reinforcers, using the tablet?	Getting more free time	Getting the tablet	Working with my friend

**Table 4:** Social validity scores for the RBTs.

Item	Erica	Summer	Lauren	Lillian
This intervention was easy to implement.	5	5	4	5
This intervention was easy to teach to students.	4	3	4	3
This intervention did not take much time away from class.	5	5	5	4
This intervention helped my student(s) stay focused on their tasks.	5	5	5	5
I liked using this intervention in my classroom.	5	5	5	4
I will use this intervention again in the future.	5	5	5	4
Mean Scores	4.8	4.7	4.7	4.2

**Table 5:** Social validity scores for the peer mediator.

Question	Ricky	Joe
Helping my friend in class was easy.	5	5
Helping my friend in class did not distract me from completing my own tasks.	5	3
I would help my friend again in the future.	5	5
I think the tablet helped my friend do their work.	5	3
Mean Scores	5	4
What did you like most—helping your friend, helping your RBT, receiving a reinforcer?	Helping my friends	Earning my choice (reinforcer)

## Data Availability

Data for this study were collected by the first author in partial fulfillment of the requirements for the Master of Arts degree in Applied Behavior Analysis at the University of South Florida.
